# Supplementary material for: Targeting the SREBP-1/Hsa-Mir-497/SCAP/FASN Oncometabolic Axis Inhibits the Cancer Stem-like and Chemoresistant Phenotype of Non-Small Cell Lung Carcinoma Cells
Source: Int J Mol Sci. 2022 Jun 30;23(13):7283. doi: 10.3390/ijms23137283 (PMC9266857; doi:10.3390/ijms23137283)
Supplement: Supplementary file 1 [file ijms-23-07283-s001.zip › ijms-1736935-supplementary.pdf]

SUPPLEMENTARY INFORMATION

Targeting the SREPB-1/Hsa-Mir-497/SCAP/FASN Oncometabolic Axis Inhibits the Cancer Stem-Like and Chemoresistant Phenotype of Non–Small Cell Lung Carcinoma Cells

Tung-Yu Tiong [1,2,] Pei-Wei Weng [3,4,5,] Chun-Hua Wang [6,7,] Syahru Agung Setiawan [8,9,] Vijesh Kumar Yadav [9,] Narpati Wesa Pikatan [10,] Iat-Hang Fong [9,] Chi-Tai Yeh [9,11,] Chia-Hung Hsu [12,13,14*], Kuang-Tai Kuo [1,2*]

1 Division of Thoracic Surgery, Department of Surgery, School of Medicine, College of Medicine, Taipei Medical University, Taipei 110, Taiwan; 09262@s.tmu.edu.tw

2 Division of Thoracic Surgery, Department of Surgery, Taipei Medical University—Shuang Ho Hospital, New Taipei City 235, Taiwan.

3 Department of Orthopaedics, School of Medicine, College of Medicine, Taipei Medical University, Taipei 11031, Taiwan; wengpw@tmu.edu.tw.

4 Department of Orthopaedics, Shuang Ho Hospital, Taipei Medical University, New Taipei City 23561, Taiwan

5 Graduate Institute of Biomedical Materials and Tissue Engineering, College of Biomedical Engineering, Taipei Medical University, Taipei 11031, Taiwan.

6 School of Medicine, Buddhist Tzu Chi University, Hualien 970, Taiwan.

7 Department of Dermatology, Taipei Tzu Chi Hospital, Buddhist Tzu Chi Medical Foundation, New Taipei City, 231, Taiwan.

8 International Ph.D. Program in Medicine, College of Medicine, Taipei Medical University, Taipei City 110, Taiwan.

9 Department of Medical Research & Education, Taipei Medical University—Shuang Ho Hospital, New Taipei City 235, Taiwan.

10 Division of Urology, Department of Surgery, Faculty of Medicine, Universitas Gadjah Mada, Yogyakarta 55281, Indonesia.

11 Department of Medical Laboratory Science and Biotechnology, Yuanpei University of Medical Technology, Hsinchu 300, Taiwan.

12 Department of Emergency Medicine, Shuang-Ho Hospital, Taipei Medical University, New Taipei City 235, Taiwan.

13 Graduate Institute of Injury Prevention and Control, College of Public Health, Taipei

Medical University, Taipei City 110, Taiwan.
14 Department of Emergency Medicine, School of Medicine, Taipei Medical University, Taipei 110, Taiwan.

* Corresponding Author

Kuang-Tai Kuo, MD., PhD
Division of Thoracic Surgery, Department of Surgery, School of Medicine, College of Medicine, Taipei Medical University, Taipei 110, Taiwan. Phone: 886-2-2490088 ext. 8881; FAX: 886-2-2248-0900. E-mail: ktkuo@tmu.edu.tw

Chia-Hung Hsu, MD
Department of Emergency Medicine, Shuang-Ho Hospital, Taipei Medical University, New Taipei City, Taiwan; Phone: 886-2-2490088 ext. 2919. FAX: 886-2-2248-0900. E-mail: 12119@s.tmu.edu.tw

Running title: SREPB-1/Hsa-Mir-497/SCAP/FASN axis as a therapeutic strategy for NSCLC

Supplementary Table S1.

No.	Target	Dilution	MW (kDa)	Source	
1	SREBP-1	1:1000	185	Cat. # ab3259	Abcam
2	SCAP	1:1000	185	Cat. # ab190103	Abcam
3	INSIG1	1:1000	185	Cat. # ab70784	Abcam
4	Vimentin	1:1000	57	#5741	Cell Signaling
5	N-Cadherin	1:1000	140	#13116	Cell Signaling
6	KLF4	1:1000	60	ab129473	Abcam
7	NANOG	1:1000	37	ab109250	Abcam
8	OCT4	1:1000	45	ab200834	Abcam
9	CD133	1:1000	110	ab222782	Abcam
10	FASN	1:1000	85	Cat. # ab128870	Abcam
11	GAPDH	1:1000	33	Cat. # ab8245	Abcam
12	HMGCS1	1:1000	33	Cat. # ab155787	Abcam
13	β-actin	1:10000	42	66009-1-Ig	Proteintech

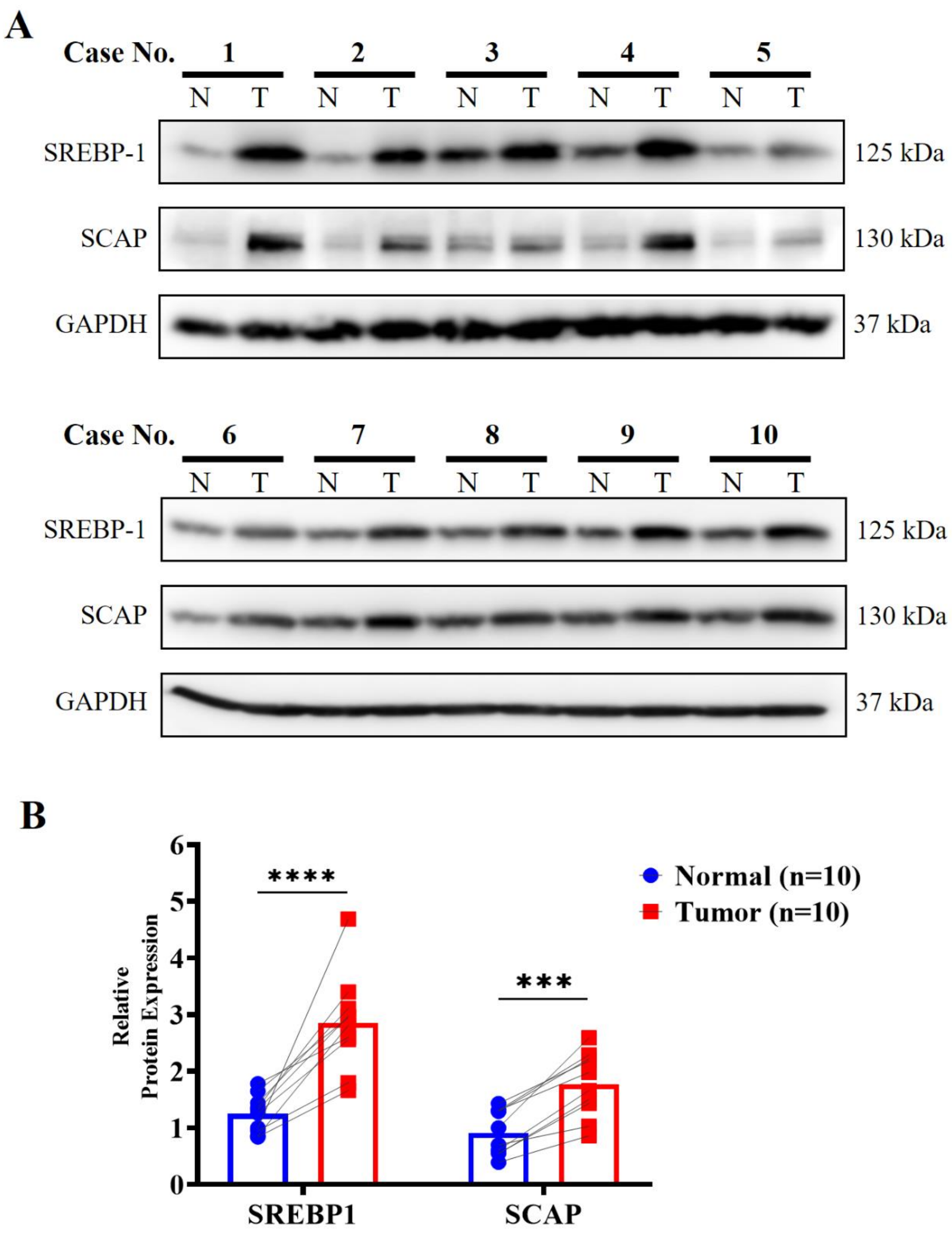

Supplementary Figure S1. Comparison of SREBP-1 and SCAP expression in tumor and normal counterparts within lung tissue specimens (*n*=10). (**A**) Western blotting representative figure from each case of lung cancer specimen denoted increased expression of SREBP-1 and SCAP in tumor than normal site of lung cancer. (**B**) Bar plot represented elevated expression of both SREBP-1 and SCAP in tumor than the normal parts of 10 cases lung cancer patients. ***p < 0.001, and ****p<0.0001.

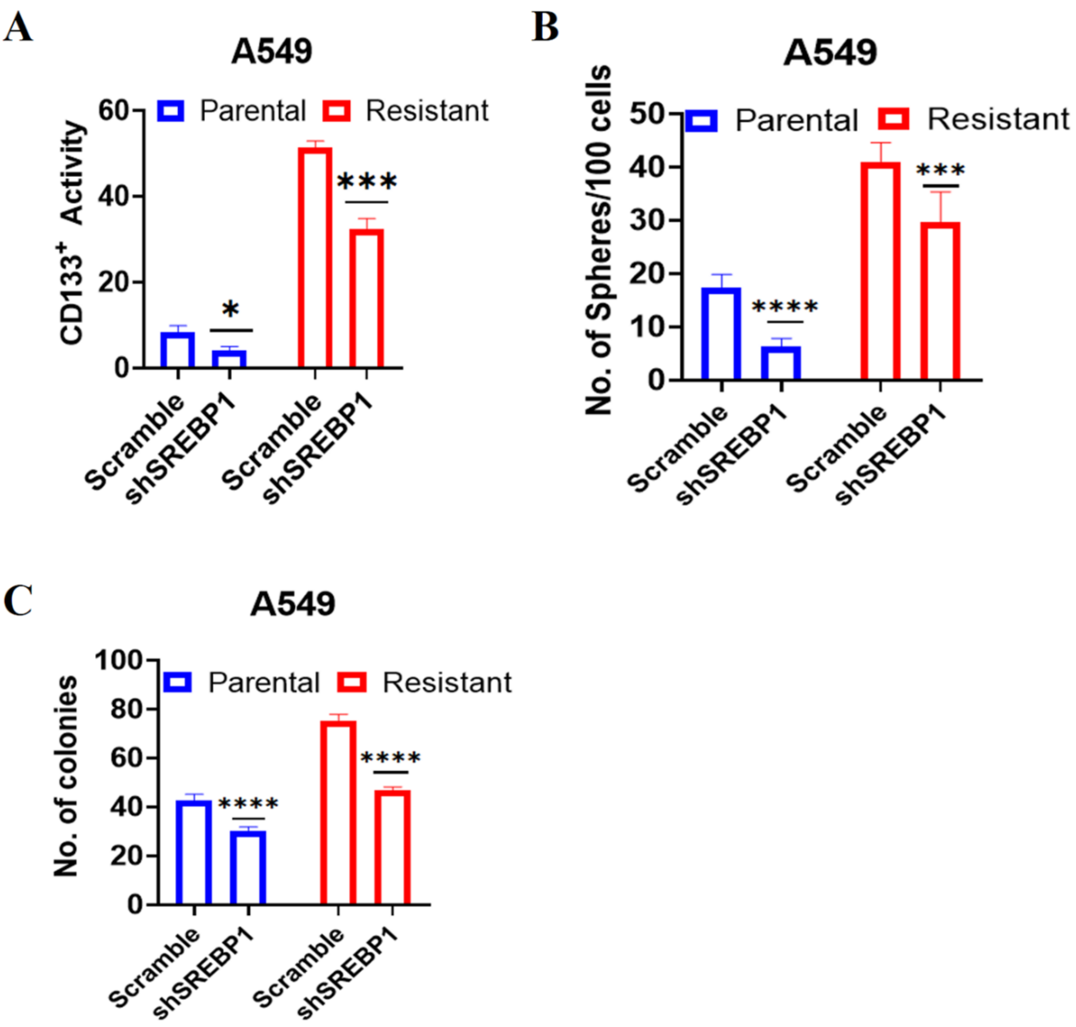

Supplementary Figure S2. Loss of SREBP-1 suppresses cancer stemness phenotypes of both parental and resistant clone of A549 NSCLC cell line. (A) Bar plot denoted attenuation of CD133+ activity in shRNA-mediated knock down of SREBP-1 of A549 cell. (B) Bar chart represented suppression of sphere formation following deficiency of SREBP-1 in both parental and resistant A549 cells. (C) Diagram signified reduction of colony forming capacity after inhibition of SREBP-1 of A549 cells, in both parental and resistant clone. *$p < 0.05$, ***$p < 0.001$, and ****$p<0.0001$.

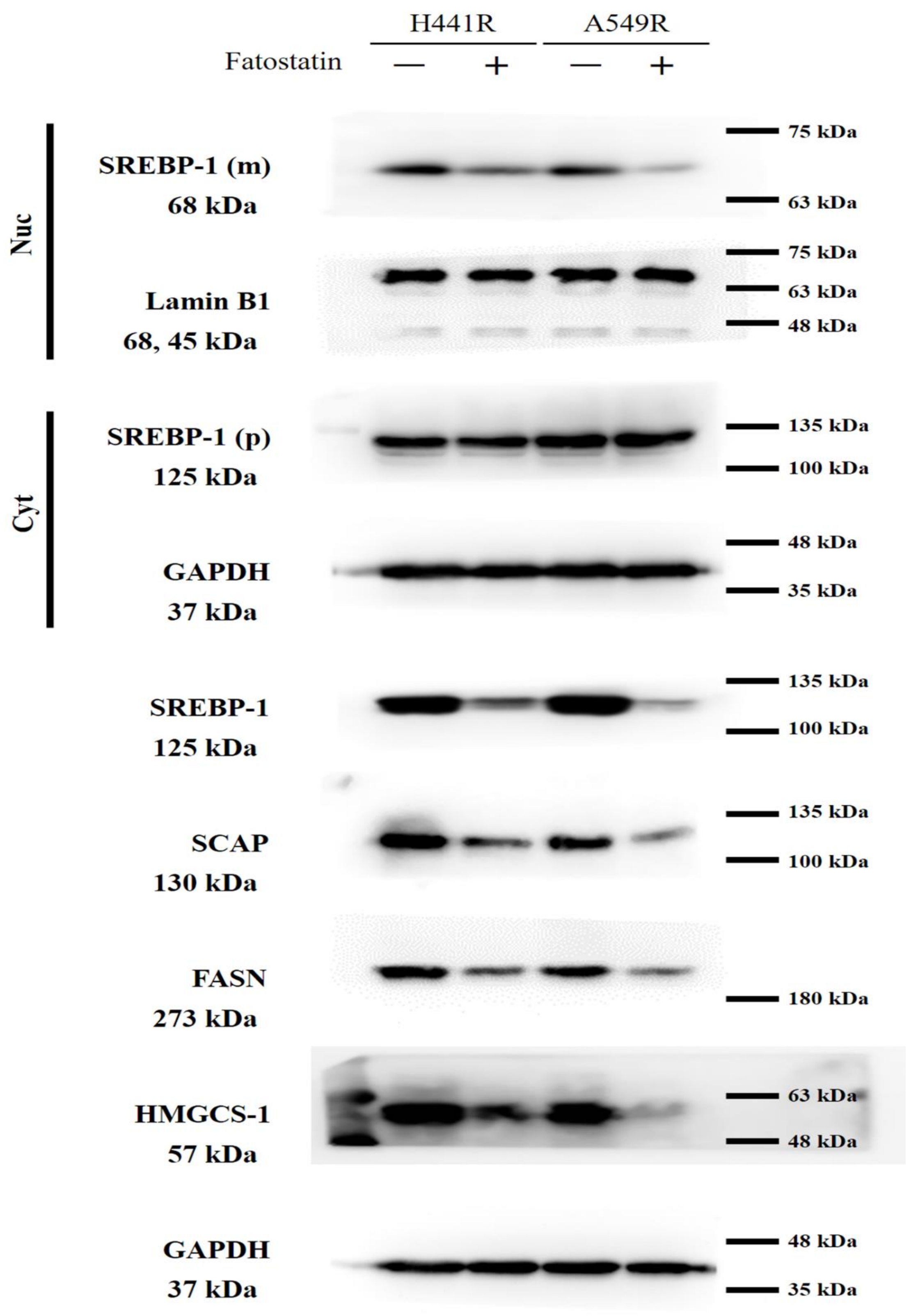

Supplementary Figure S3. Full-size blots of Figure 3B

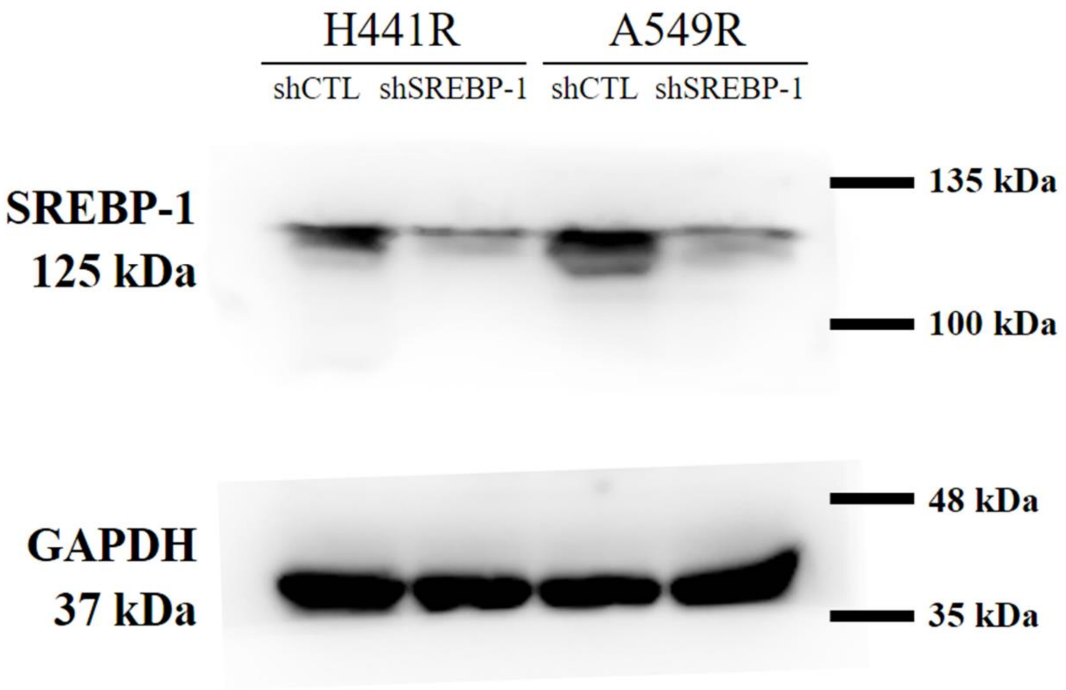

Supplementary Figure S4. Full-size blots of Figure 4A

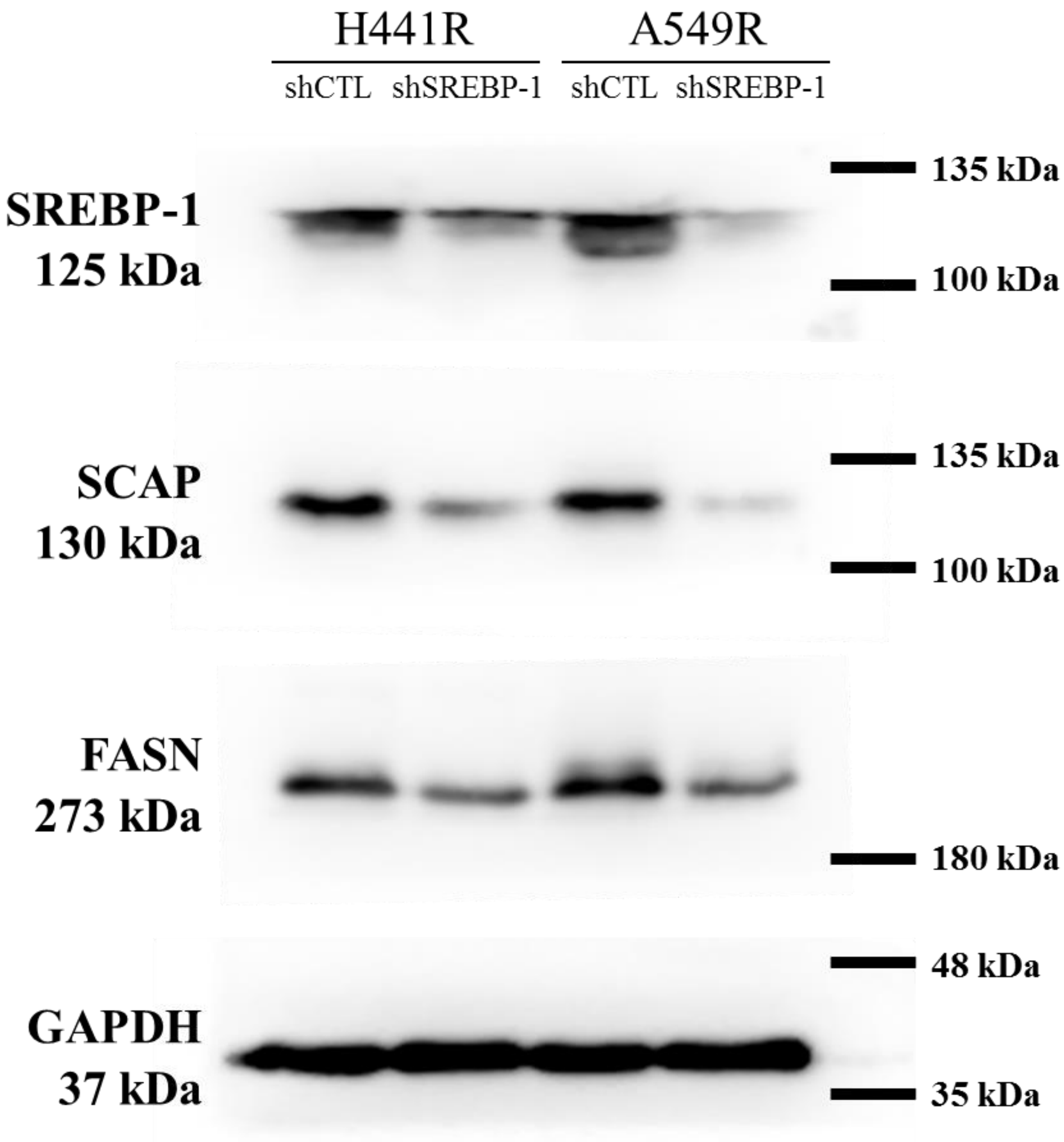

Supplementary Figure S5. Full-size blots of Figure 4D

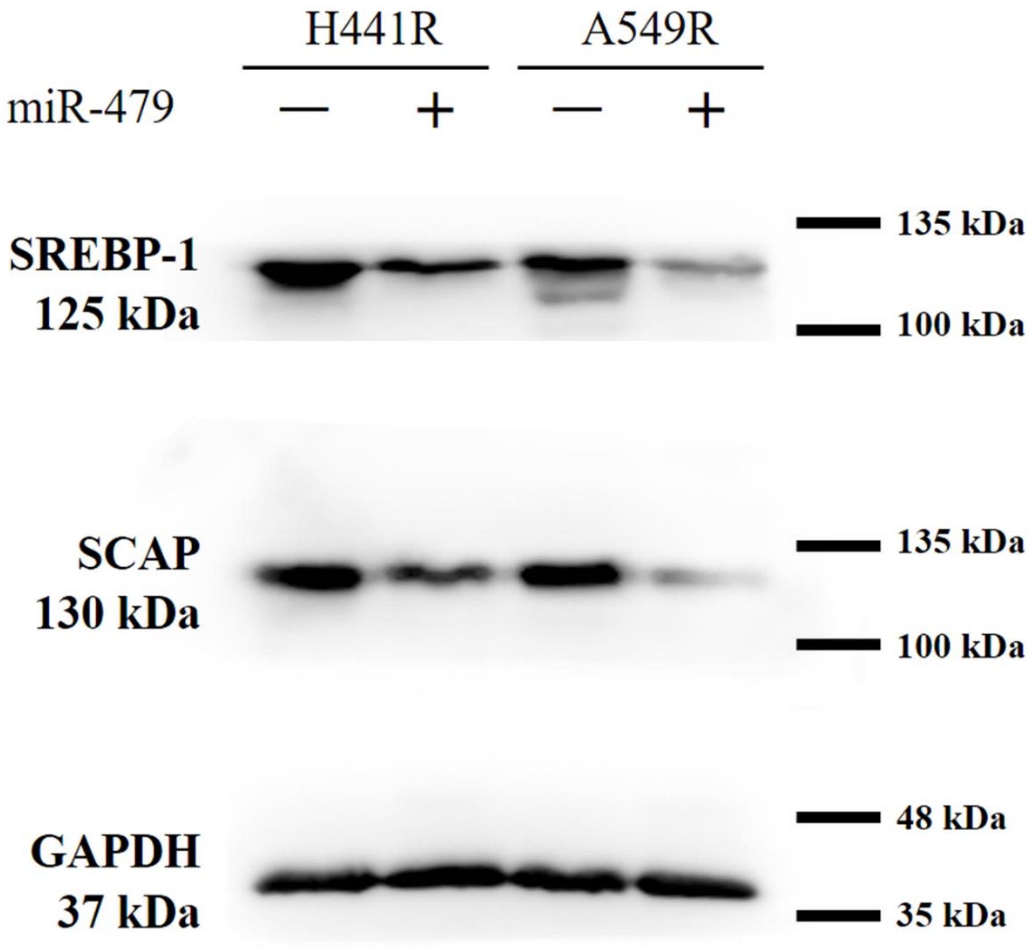

Supplementary Figure S6. Full-size blots of Figure 6E

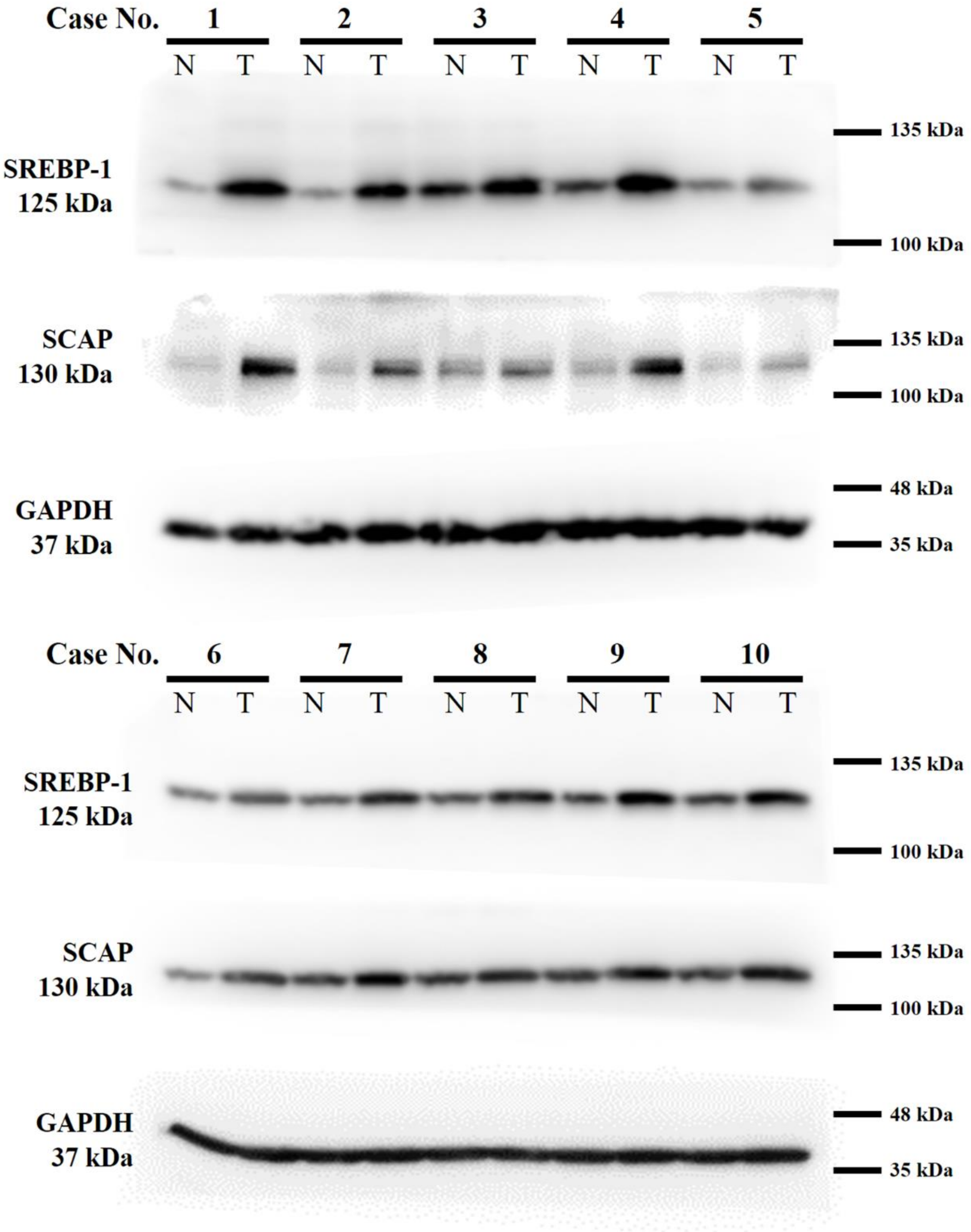

Supplementary Figure S7. Full-size blots of Supplementary Figure S1A
